# Adaptations of a Tertiary Otorhinolaryngology Head and Neck Surgery Department in Singapore during the COVID-19 Outbreak

**DOI:** 10.1177/0003489420946779

**Published:** 2020-07-29

**Authors:** Anna See, Lih Khuang Go, Constance E. H. Teo, Neville Wei Yang Teo, Song Tar Toh

**Affiliations:** 1 Department of Otorhinolaryngology Head and Neck Surgery, Singapore General Hospital, Singapore

**Keywords:** infection control, pandemic, Otolaryngology, endoscopy, coronavirus

## Abstract

**Purpose:**

The novel coronavirus 2019 (COVID-19) outbreak which was first reported in Wuhan, China has been declared a pandemic by the World Health Organization on March 11, 2020. Otorhinolaryngologists deal intimately with pathologies of the head and neck region and upper respiratory tract and have been reported as a vulnerable group of healthcare workers who may be more susceptible to COVID-19 nosocomial infection.

**Methods:**

In this article, we provide a comprehensive overview of the adaptations of Singapore’s largest tertiary Otorhinolaryngology department during the COVID-19 outbreak. This was undertaken via an evidence-based approach. The relevant medical literature and evidence underlying our adaptations are highlighted.

**Results:**

A four-pronged strategy including (1) personnel segregation, (2) triaging and decantment, (3) use of personal protective equipment and (4) changes in clinical practice was employed. The strategy was bolstered by drawing upon a collective learnt experience from the 2003 Severe Acute Respiratory Syndrome (SARS) outbreak.

**Conclusion:**

A rigorous framework which can preserve operationality while navigating the heightened risks during this outbreak is critical for every Otorhinolaryngology department. As the pandemic continues to evolve and more scientific reports of this disease are made available, approaches will need to be morphed.

## Introduction

The Coronavirus Disease 2019 (COVID-19) outbreak was declared by the World Health Organization to be a Public Health Emergency of International Concern on January 30, 2020, and a pandemic on March 11, 2020.^1^ Singapore reported her first human case of COVID-19 infection on January 23, 2020. Singapore has a land area of 721.5 km^2^ and is served by less than 20 acute hospitals. As of May 31st, 2020, this small city-state with a population of 5.6 million, has a total of 34,884 confirmed cases and 23 deaths.^2^

Rapidly accumulating reports identify otolaryngologists as high-risk healthcare workers (HCW) who are more susceptible to the COVID-19 infection due to the anatomy we treat. The first documented physician deaths due to COVID-19 nosocomial infection in Wuhan and the United Kingdom’s National Health Service (NHS), were both that of otolaryngologists.^3,4^

In this article, we report the evidence-based adaptations of Singapore’s oldest and largest tertiary Otorhinolaryngology Head and Neck Surgery department in preserving operationality while navigating the heightened risks during this outbreak. This was carried out via a four-pronged approach, taking into consideration our institution’s experience from the 2003 Severe Acute Respiratory Syndrome (SARS) outbreak.

## Four-Pronged Approach

### 1) Personnel Segregation

During the 2003 SARS outbreak, Singapore reported the world’s second highest rate of HCW infection at 41% (93 out of 234 total cases), just short of Canada’s 43%.^5^ SARS’s high HCW infection rate and subsequent mortality highlighted the importance of preventing HCW inter-transmission.^6,7^ In the current pandemic, we adopted a strategy of immediate segregation into teams to limit HCW interaction and to allow for essential clinical services to continue in the event one team was quarantined due to a team member infection. This also allows for efficient contact-tracing should an unprotected exposure event occur.

The department was divided into 2 teams comprising of otolaryngologists of varying seniority. Each team comprised of Senior Consultants, Consultants, Registrars and Trainees. The subspecialties of Otology, Head & Neck Surgery, Sleep Surgery and Rhinology were represented in each grouping.

Elimination of crowds and social distancing have been advocated as the most effective measures to dampen the surge in cases.^8,9^ As such, two distinct clinical areas distanced at least 200 meters apart were assigned for outpatient practice since February 2020. Location A was designated as the “Active” clinical area and the assigned team oversaw the week’s emergency calls, inpatient care, surgeries and outpatient follow-up. Location B was designated as the “Passive” clinical area and its assigned team attended to a reduced number of new patient referrals, with their activities limited only to Location B. Disinfection of both areas was performed daily and each week, the teams swapped locations and duties. Patients requiring follow-up were instructed to report to the correct location for review by the same team of doctors. Strict segregation was enforced, and each team was discouraged from meeting professionally or socially.

In anticipation of referrals for otolaryngologic assessment and/or tracheostomies of confirmed COVID-19 cases, a single Senior Consultant from the “Active” team each week was placed on a week-long call duty. Recommendations on minimizing risk of viral transmission during tracheostomies were reviewed regularly and each of the senior staff members rostered on-call has been a practicing medical professional since the 2003 SARS outbreak.^10,11^ This was in accordance with recommendations to have the most skilled and/or experienced surgeon for the procedure.^12^ To minimize the personnel exposed to confirmed cases, junior staff were relieved of this duty.

In the initial phase of the outbreak, all department activities such as weekly morbidity and mortality (M&M), grand rounds and lectures were suspended. In the tenth week, recognizing that this outbreak was a prolonged battle, a new normalcy was sought with resumption of departmental activities. M&M meetings, multidisciplinary tumor boards and teaching sessions were hosted via webcast facilities such as Webex and Zoom. Both teams participated at distinct physical locations.

### 2) Triaging and Decantment of Patients

**Figure 1.**
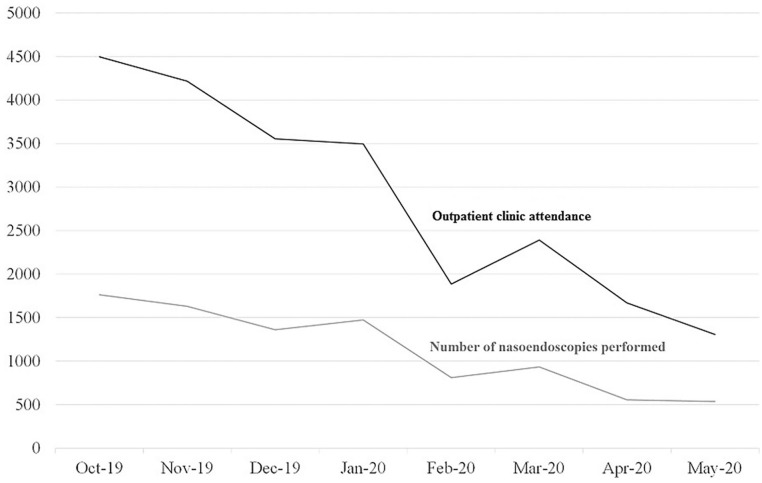
Monthly statistics for the department’s outpatient clinic attendance (black line) and nasoendoscopy numbers (grey line) since October 2019 till May 2020.

A list of criteria was generated to identify non-urgent conditions and all scheduled clinic appointments were screened two weeks prior. Patients with non-urgent conditions were telephone-consulted and rescheduled minimally six months later, with prescriptions written for delivery. Follow-up for malignancies and immediate postoperative cases was prioritized. Figure 1 shows the department’s monthly outpatient clinic attendance and the number of nasoendoscopies performed from three months prior to the outbreak, October 2019, until May 2020. Singapore’s first case of COVID-19 infection was confirmed on 23 January 2020. Since February 2020 till May 2020, the department has managed a total of 7250 outpatient attendances and performed 4308 nasoendoscopies with zero HCW nosocomial infection.

Patients were allowed into clinical areas only after triage and a comprehensive questionnaire, which details the presence of any respiratory symptoms, travel history and contact history. In recent months, anosmia and dysgeusia have been reported as possible symptoms of COVID-19 infection.^13-15^ Our institution investigated the sensitivity and specificity of self-reported olfactory and taste dysfunction (OTD) as a screening question for COVID-19 infection and found that it had a high specificity as a screening criterion.^16^ This symptom has since been included at our triage. Within each clinical area, an enclosed room is set up as an “Isolation Consult Room.” Patients who are suspected to harbour COVID-19 infection due to affirmative answers in the questionnaire are temporarily isolated in the room. The attending Otolaryngologist is then alerted of the case and must see the patient in the “Isolation Consult Room.” If a similar high index of suspicion is held after the Otolaryngologist’s review, the patient is sent to the Emergency Department for an immediate COVID-19 respiratory swab. Since the implementation of the “Isolation Consult Room” in end January 2020, we successfully identified two patients with COVID-19 infection, whose index presentation was to the outpatient clinic. One, in a patient with anosmia and low-grade fever, and another, in a patient with hoarseness and no fever. This system prevented mingling of these two suspect cases with the other patients who were awaiting consultation in the common area, and also limited the number of HCWs exposed to them. Interaction was performed in full personal protective equipment including disposable surgical gown, hair cap, eye protection, N95 respirator mask and surgical gloves. The suspect cases were ultimately confirmed to have COVID-19 infection via a positive respiratory swab for SARS-CoV-2.

Our department performs an average of 40 elective surgeries a week and this number has halved since the outbreak. Only a single team of doctors operates each week. Surgeries were allowed to proceed if they were of the following nature: emergency or life-saving, suspected or known malignancy and/or procedures not requiring admission. The last criterion ensured that hospital occupancy was not affected, and beds remained available for COVID-19 admissions.

### 3) Use of Personal Protective Equipment

Personal Protective Equipment (PPE) is a precious resource in major disease outbreaks. As otolaryngologists are at high risk of contact with respiratory droplets or aerosolized upper airway particles, the need for a sustainable and evidence-based PPE usage guideline was of paramount importance. At present, the causative pathogen for COVID-19, SARS-CoV-2, is believed to be transmitted via respiratory droplets.^17^ While various guidelines have been released, there is currently no international consensus on the best method of reducing the risk of virus transmission.

In our department, the following was implemented at the initial phase of the outbreak:

- In outpatient clinics, a surgical mask was required for all patient encounters. If a nasoendoscopy was to be performed, a fitted N95 mask and eye protection (either faceshield or surgical goggles) were to be worn.
- In operating theatres, a surgical mask was required for all surgeries. If the surgery involved mucosa of the upper airway, a fitted N95 mask and eye protection were to be worn. For any surgery involving confirmed COVID-19 cases, a Powered Air-Purifying Respirator (PAPR) was to be worn.

As the outbreak intensified and medical literature expanded, debates on the mode of viral transmission and the type of PPE required, grew.^18-20^ In the tenth week of the outbreak, after expansive literature review and discussion with Infectious Disease specialists, the department guidelines were modified to:

- In outpatient clinics, a surgical mask was required for all patient encounters. If a nasoendoscopy was to be performed, a shower cap, water-resistant gown, N95 mask and eye protection were to be worn.
- In operating theatres, a surgical mask and eye protection were required for all surgeries. For any surgery involving mucosa of the upper airway, minimum requirement included N95 mask and eye protection, regardless of the patient’s COVID-19 status. PAPRs were available for surgeons who felt more comfortable donning them. Functional PAPR units are available in our institution since the SARS outbreak, and further acquisition is underway. A coordinated effort was made to ensure that daily surgery listing did not exceed the availability of PAPRs.
- At the time of writing, routine pre-operative COVID-19 testing for all patients is not available in our institution. We are constantly reviewing our guidelines to ensure sustainable use of minimum PPE for surgeon safety.

### 4) Changes in Clinical Practice

#### Use of telemedicine

The use of Telemedicine was piloted in our laryngology clinics. Weekly, a videoconference was established with an off-site laryngologist. An on-site resident would perform videostroboscopies on patients and live-stream to the laryngologist before discussing management plans. This prevented disruption of laryngological services as our attending laryngologist had been deployed off-site.

#### Minimization of aerosol-generating procedures

Three aspects of our practice were identified to be potentially aerosol-generating and modifications were implemented.

- The use of energy devices such as Harmonic Scalpel during surgery^21,22^
  Modification: A reduction in use was recommended. If use was necessary, the surgeon was to don, minimally, N95 mask and eye protection even if the surgery did not involve mucosa of the upper airway.
- The use of high-speed drills and debriders during surgery^23,24^
  Modification: Non-urgent endoscopic sinus surgeries were postponed. Emergent skullbase surgeries such as those performed for pituitary decompression were allowed to proceed with PAPR.
- The use of nasal decongestant and anesthetic via a atomizing nozzle spray during nasoendoscopy^25^
  Modification: Doctors were recommended to use a sterile water-based lubricant gel for nasoendoscopy (applied over the nasoendoscope) instead of nasal sprays.

#### Simulation for emergency scenario

Within the first month of the outbreak, a flowchart was designed for emergency surgical airway creation for COVID-19 patients with acute cardiorespiratory arrest. A joint simulation exercise was carried out with Anaesthesia, Operating Theatre Staff and Emergency Department to ensure familiarity and to identify areas for improvement. To date, we have not had any COVID-19 cases requiring emergency surgical airway creation but this workflow remains highly relevant in the event of need.

## Other Measures

In addition to the unique four-pronged approach, our department also adhered to institutional (Singapore General Hospital) and national measures which included:

- Twice-daily temperature taking of all staff. Any staff with temperature ≥37.5°C (99.5°F) were mandated to report to the staff clinic for assessment.
- Cessation of cross-institution HCW movement - Visiting specialists were not allowed to practice at more than one acute hospital.
- Restriction of cross-institution patient movement – Admitted patients from another acute hospital could not be transferred without hospital management approval.

## Conclusion

The pandemic continues to evolve and changing disease trends will require constant adaptations. Through this article, we hope to have provided a comprehensive overview of the operational adaptations undertaken based on existing evidence. We wish to highlight that while panic and anxiety are inevitable in a novel viral outbreak, it is of paramount importance to stay abreast of the science and to actively adopt an evidence-based approach for departmental strategic planning. In our institution, the lessons learnt from Singapore’s SARS outbreak in 2003 are etched in the minds of many. The rapid conceptualization of a business continuity plan when this outbreak gained traction, is in large part, due to past painful experience. At the time of writing, no Otolaryngologist in our institution has been infected with COVID-19 and it is our greatest hope that this remains so. We are prepared to morph our approach as the pandemic continues to evolve locally and internationally, and as more scientific reports of this disease are made available.
